# Observing one-step melting pathway in two-dimensional hard circular particle system

**DOI:** 10.1016/j.isci.2025.113107

**Published:** 2025-07-12

**Authors:** Yanwen Zhu, Shenhua Jiang, Jieli Wang, Xin Song, Xing Huang, Bin Zheng, Linli He, Shigeyuki Komura, Zhanglin Hou

**Affiliations:** 1 Department of Physics, Wenzhou University, Wenzhou, Zhejiang 325035, China; 2 Wenzhou Institute, University of Chinese Academy of Sciences, Wenzhou, Zhejiang 325001, China; 3 Department of Mechanical Engineering, Hangzhou City University, Hangzhou 310015, China; 4 OuJiang Laboratory (Zhejiang Lab for Regenerative Medicine, Vision and Brain Health), Wenzhou, Zhejiang 325000, China; 5 Department of Chemistry, Graduate School of Science, Tokyo Metropolitan University, Tokyo 192-0397, Japan

**Keywords:** condensed matter physics

## Abstract

The nature of two-dimensional melting remains a matter of debate. Based on Langevin dynamics simulation, we present a surprising observation that the melting pathway of hard circular-particles/disks is relevant to the dynamical properties of particles. Using hard circular-particles/disks, where particle friction is proportional to particle size, results show the melting pathway of small size particles obeys two-step scenario, consistent with previous reports on melting behavior of hard disks. Conversely, in large size particle systems, we observe a one-step melting pathway. Further investigation reveals that the emergent one-step melting pathway is non-equilibrium phase transition that relates to cooperative relaxation of cage dynamics. This originates from the particularly stable/stiff configuration in large size particle system, which is a metastable state arrested by changed dynamics of particles, thus inducing a non-equilibrium melting pathway that distinguishes from the equilibrium melting behavior. Our findings shed light on the debate on melting behavior of hard disks.

## Highlights

• The melting process relates to the cooperative relaxation of cage dynamics
• Non-equilibrium one-step melting pathway is observed in 2D hard circular particle systems
• The non-equilibrium melting pathway originates from the metastable state arrested by dynamics
• The melting pathway is tunable through the size of particle

## Graphical abstract

**Figure:**
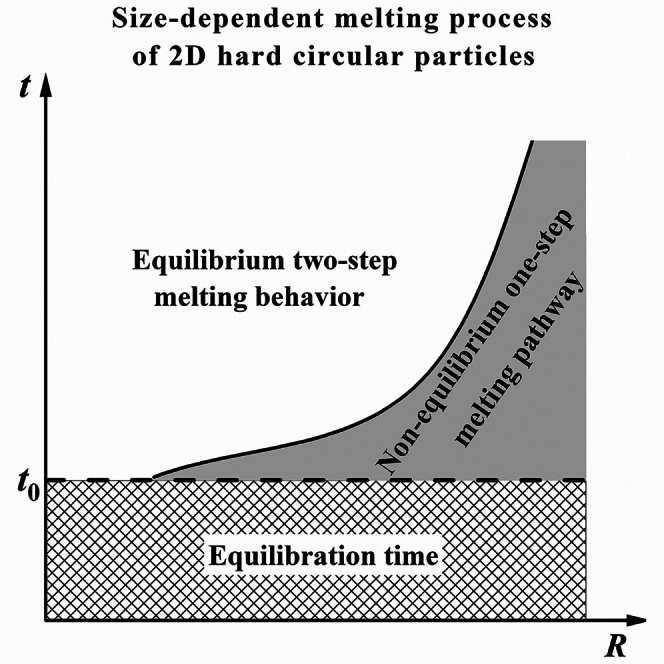

## Introduction

The nature of melting in two-dimensional (2D) systems attracts significant interest not only for its close connection with the fundamental principles of statistical physics but also for its potential applications in the industrial development of micro-nano-scale materials. However, our understanding on 2D melting remains poor, due to a lack of systematic and comprehensive investigation as well as relevant theoretical advancements. Over the past decades, extensive investigations have dedicated to 2D melting and revealed that melting of 2D systems is closely related to particle shape,^1^^,^^2^^,^^3^^,^^4^ configuration density,^5^ intermolecular interaction,^6^^,^^7^ and constitute particles in system.^8^^,^^9^ These findings reflect a non-trivial behavior of 2D melting, consistent with the concept of “more is different”.

The specific underlying mechanism of 2D melting hitherto remains elusive due to two key difficulties. The first one is the existing interplay mechanism among various competing energies, which keeps system in a stable/metastable state through a complicated but subtle balance, e.g., the competition between orientational entropy and positional entropy,^1^ conflict between particle shape and intermolecular interaction,^6^ correlation between particle dynamics and phase selection,^10^ and competition between diverse local polymorphic configurations that are formed by neighboring particles.^11^ The other main difficulty arises from the problem in determining whether the system is at equilibrium or not. This issue is particularly significant in the physics of melting. In general, an equilibrium system is in the state of minimum free energy, however, how to identify/approach the energy minimum state is a critical issue, depending on various degrees of freedom, such as the polydispersity,^12^ shapes,^13^^,^^14^ and interactions^15^ of particles, and the dynamical processes of systems.^16^^,^^17^^,^^18^^,^^19^

Actually, the equilibrium state of some systems is not always realized. Previous investigations revealed that it is possible to trap a system at a metastable non-equilibrium state through various approaches, which include rapid quench operations,^19^ introducing a short-range attractions (protein systems), applying a small amount of uniaxial stress,^20^ tuning the size or volume fraction of depletion agents^18^ and shape-designing of constitute particle.^11^ Such techniques can result in a non-equilibrium phase transition pathway that distinguishes from the equilibrium phase behavior. It is known that the dynamics of particles will not influence the equilibrium phase behavior of system, but how they affect the relaxation process before system reaches equilibrium remains an open question. This issue is crucial not only for understanding the mechanism of equilibrium/non-equilibrium phase transition, but also for improving the efficiency of equilibrium process that matters to facilitate industrial applications.

In this work, based on Langevin dynamics simulation, we aim to investigate the effect of dynamics on the melting phase transition in 2D hard circular-particle system. Inspired by the previous experimental studies^21^^,^^22^ where the motion of particle can be controlled by internal activity through a complex (i.e., yolk-like) structural design, we try to control the dynamics of hard circular-particles by adjusting certain degrees of freedom, e.g., introducing internal activity of particles, to investigate the melting process of system. Specifically, we use Lennard-Jones beads to construct a yolk-like particle, which is composed of a radius-tunable ring and a constant-radius core. In small size particle systems, we observe an equilibrium two-step melting behavior, consistent with previous reports on hard disk systems.^23^^,^^24^^,^^25^ Strikingly, in the large size particle systems, we observe a non-equilibrium one-step melting pathway. We reveal that the emergent one-step melting pathway in large size particle systems is related to a cooperative relaxation of cage dynamics. Furthermore, by using a simple hard disk model, we stress that this observation is universal.

### Model

**Figure 1:**
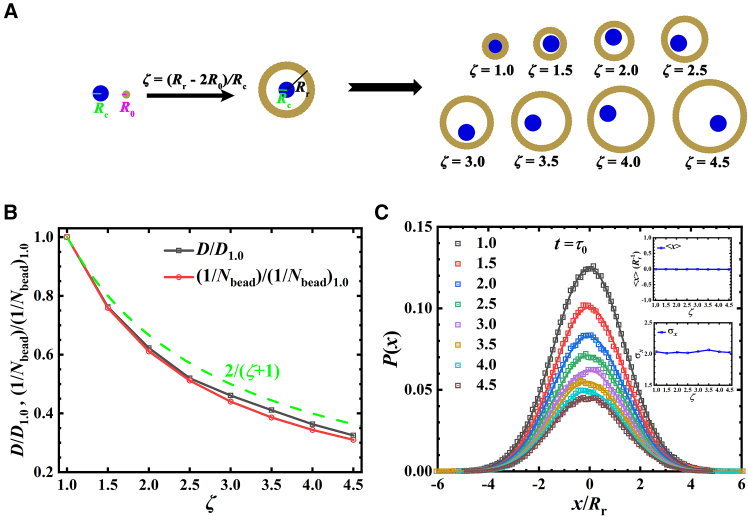
Sketch of yolk-like particle and its single-particle dynamics properties (A) The yolk-like particle is composed of two parts: a constant-radius (*R*_c_) core and a radius-tunable (*R*_r_) ring. The core is a hard disk (blue) with radius of *R*_c_ = 2*R*_0_, where *R*_0_ = 2^(−5/6)^*σ*. The ring consists of hard Lennard-Jones beads (gold) with radius of *R*_0_. These beads are put on a radius of *R*_r_-*R*_0_, and the radius of ring *R*_r_ is controlled by *ζ*=(*R*_r_-2*R*_0_)/*R*_c_. The particles with different *ζ* used in simulations are shown in the right panel of (a). (B) The relative diffusion constants (normalized by *ζ* = 1.0 case) as a function of *ζ*, the diffusion constant obtained from single-particle mean-square displacement (MSD). The green dashed line is the theoretical prediction obtained from Einstein relation. (C) The distribution probability of displacement on *x* axis of each *ζ* particle at *t* = *τ*_0_. *τ*_0_ is the timescale that a particle diffuses one particle length scale, i.e., $\left\langle \Delta r^{2}\left(\tau_{0}\right)\right\rangle =4R_{r}^{2}$. The solid lines are Gaussian fittings. Insets are the average value and variance of distribution.

We use two kinds of hard Lennard-Jones (LJ) beads with radii of *R*_0_ = 2^(−5/6)^σ and *R*_c_ = 2*R*_0_ to build a yolk-like particle, as shown in Figure 1A, where σ is the unit of length in LJ system. The *R*_0_-radius beads are arranged to form a ring at the radial distance of *R*_r_-*R*_0_, where *R*_r_ is the radius of particle/ring, and *R*_c_-radius beads are put inside this ring. We define *ζ*=(*R*_r_-2*R*_0_)/*R*_c_ to control the particle radius, and particles with various *ζ* are shown in Figure 1A. See details in STAR Methods.

We use a GPU-based molecular dynamics package, the GPU-accelerated large-scale molecular simulation toolkit (GALAMOST)^26^^,^^27^ to perform Brownian dynamics simulations of particles. In principle, the dynamics of particles are modeled by Langevin dynamics. The velocity ***v***(*t*) obeys the following equation

(Equation 1) $$m\dot{v}\left(t\right)=-\gamma v\left(t\right)+F\left(t\right)+F_{r}\left(t\right)\text{,}$$

where ***F***(*t*) is the total force exerted on a particle through the interactions with neighboring particles, and *γ* is the friction constant of particle. Here, *γ* = *N*_bead_∗*γ*_bead_ with *N*_bead_ the number of beads on a rigid body and *γ*_bead_ the friction constant per bead. Note that the core and the ring are the components of one particle, but they are treated as independent rigid bodies in simulations. Moreover, $F_{r}\left(t\right)=\sqrt{2\gamma_{\text{bead}}k_{B}T}\sum_{i=1}^{N_{\text{bead}}}\xi_{i}\left(t\right)$ is the random force, where $\xi_{i}\left(t\right)$ is the normalized Gaussian white noise acting on bead *i*, *k*_B_ is the Boltzmann constant and *T* is the temperature of the system. With an external thermal bath, the ring and core fluctuate independently while interact with each other through collisions, see Video S1 of a representative motion of *ζ* = 4.5 particles.

Video S1. Representative motion of ζ=4.5 particles, related to Figure 1

## Results

### Single particle dynamics and collective melting behaviors

We focus on the behavior of the particle ring (hard circular-particle) in current investigation. To analyze the single particle dynamics, we calculate the mean-square displacement (MSD), and obtain the diffusion constant as well as the probability distribution of displacement for each *ζ* particle. The diffusion constant is derived from $\left\langle \Delta r^{2}\left(t\right)\sigma^{2}/R_{0}^{2}\right\rangle =4Dt$. Additionally, considering the size of each *ζ* particle, the effective diffusion constant is calculated by using $\left\langle \Delta r^{2}\left(t\right)\sigma^{2}/R_{r}^{2}\right\rangle =4D_{\text{eff}}t$, which will be discussed further. Figure 1B shows the relative diffusion constant (normalized by that of *ζ* = 1.0 case) as a function of *ζ*. In principle, the diffusion constant obeys the Einstein relation, *D* = *k*_B_*T*/*γ*. Given that *γ* is the summation of all beads, *D* is inversely proportional to *N*_bead_ (red line in Figure 1B, also see *N*_bead_ for each *ζ* particle in Table S1 in supplemental information (SI)). Further, since the beads are distributed on particle evenly with the distance between two adjacent beads fixed at approximately 0.5*σ*, the friction constant of particle is roughly proportional to its radius. Consequently, the relative diffusion constant also follows the prediction $D/D_{1.0}={\left(R_{r}\right)}_{1.0}/R_{r}=2/\left(\zeta +1\right)$, where *D*_1.0_ and (*R*_r_)_1.0_ are the diffusion constant and radius of *ζ* = 1.0 particle, respectively. The displacement distributions along *x* axis at *t* = *τ*_0_ (the timescale for a particle diffusing over its own length scale, see *τ*_0_ of each *ζ* particle in Table S2) are shown in Figure 1C, following a Gaussian distribution with the average value 0 (upper inset) and a similar variance (lower inset). These tests demonstrate the particles meet the reality: the friction constant is proportional to the particle radius and their single particle diffusion behaviors show a similar feature due to isotropic nature (hard-disk-like) of particles.

**Figure 2:**
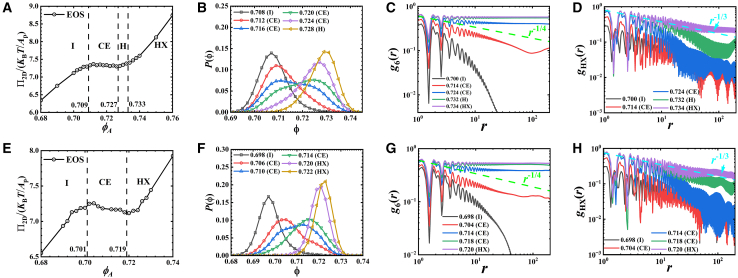
The melting pathways of *ζ* = 1.0 and *ζ* = 4.5 particles Results are obtained after 6×10^7^ and 1.5×10^8^ MD steps for *ζ* = 1.0 and *ζ* = 4.5 systems, respectively. The equation of state (EOS) (A and E), local density distribution *P*(ϕ) (B and F), 6-fold bond-orientational correlation function *g*_6_(*r*) (C and G), and spatial correlation function of hexagonal order *g*_HX_(*r*) (D and H) of *ζ* = 1.0 and 4.5 systems. Green dashed lines in (C) and (G) represent curves proportional to *r*^−1/4^, and cyan dashed lines in (D) and (H) correspond to curves proportional to *r*^−1/3^.

Although all particles show a similar single particle diffusion behavior, we observe distinct melting pathways in high and low *ζ* systems after an adequate relaxation of systems (6×10^7^/1.5×10^8^ MD steps run, more than 270 times of the characteristic timescale *τ*_0_). Figure 2 presents the observed melting pathways for particles with *ζ* = 1.0 (Figures 2A–2D) and *ζ* = 4.5 (Figures 2E–2H). For *ζ* = 1.0 particles, the melting process follows a two-step pathway. System first melts from a hexagonal crystal (HX) phase to an intermediate hexatic (H) phase continuously, and subsequently from H to isotropic fluid (I) phase by a first-order transition, characterized by a coexistence (CE) window (0.709<*ϕ*_*A*_ < 0.727, Figure 2A, *ϕ*_*A*_ = *NA*_p_/*L*_*x*_/*L*_*y*_, where *A*_p_ is the area of particle, *L*_*x*_ and *L*_*y*_ are the lengths of box along *x* axis and *y* axis, respectively). In contrast, the observed melting pathway of *ζ* = 4.5 particles exhibits a first-order one-step melting scenario, changing from HX phase to I phase directly. This is confirmed by the evidences of CE of HX and I (0.701<*ϕ*_*A*_ < 0.719, Figure 2F) as well as the Mayer-Wood loop^28^ in the equation of state (EOS, Figure 2E). In both systems, the quasi-long-range order of HX phase obeys the prediction of Berezinskii-Kosterlitz-Thouless-Helperin-Nelson-Young (BKTHNY) theory.^29^^,^^30^^,^^31^^,^^32^^,^^33^^,^^34^^,^^35^ The spatial correlation of positional order *g*_HX_(*r*) shows a power-law decay with *g*_HX_(*r*)∼*r*^−1/3^ corresponding to the stability criterion of HX phase. Similarly, the decays of bond-orientational correlation shows three regimes: long-range order in HX phase (*g*_6_(*r*)∼constant), quasi-long-range order in H phase (power-law decay) and short-range order in I phase (exponential decay).

### Cooperative relaxation related melting pathway

**Figure 3:**
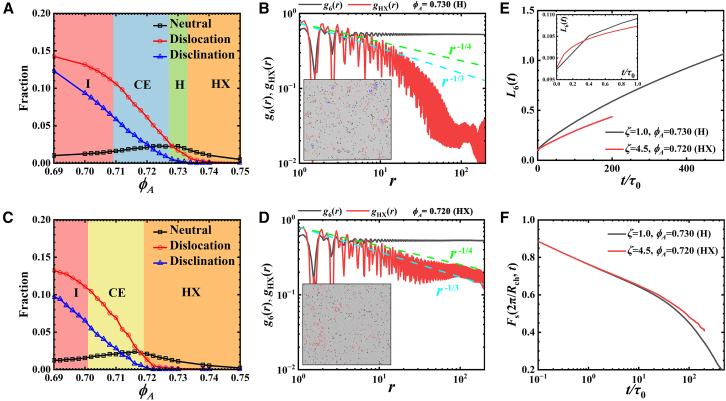
Analysis on emerging melting pathways of *ζ* = 1.0 and *ζ* = 4.5 particles (A and C) Fractions of defects as a function of *ϕ*_*A*_ in *ζ* = 1.0 (A) and 4.5 (C) systems. The fractions are calculated by taking the ratio of the number of particles involved in each type of defect and the total number of particles of the system. The colored backgrounds indicate the different phase windows. (B and D) The spatial correlation functions of bond-orientational order *g*_6_(*r*) and hexagonal crystal order *g*_HX_(*r*) of selected representative configurations in *ζ* = 1.0 (B) and 4.5 (D) systems. Insets are the configurations with defects colored: black, neutral; red, dislocation; blue, disclination. (E) The time evolution of Lindemann parameter *L*_6_(*t*) of H phase in *ζ* = 1.0 system (*ϕ*_*A*_ = 0.730) and HX phase in *ζ* = 4.5 system (*ϕ*_*A*_ = 0.720), respectively, which represents the cage dynamics in system. Inset: an enlarged view within short timescale. (F) Self-part intermediate scattering function (self-ISF) *F*_s_(2π/*R*_ch_, *t*) of H phase in *ζ* = 1.0 system (*ϕ*_*A*_ = 0.730) and HX phase in *ζ* = 4.5 system (*ϕ*_*A*_ = 0.720), respectively, where *R*_ch_ is the length scale of the first peak of radial distribution function.

Based on the BKTHNY theory, we investigate the mechanism of the melting pathways in *ζ* = 1.0 and 4.5 systems through the evolution of topological defects during melting process.^1^^,^^2^ For *ζ* = 1.0 system (Figure 3A), within high density region in HX regime, only the neutral defect is observed, which does not destroy the quasi-long-range order of HX phase. System melts to the H phase until the appearance of dislocation as density decreases (*ϕ*_*A*_ ∼ 0.733). By further decreasing the density, the amount of dislocation grows and disclination starts to appear around the peak of neutral defects (*ϕ*_*A*_ ∼ 0.727), leading the system into the CE regime. After an almost linear growth in CE window, accumulated disclinations drive the system to melt into the I phase. The melting pathway of *ζ* = 1.0 particles is closely related to the evolution of dislocations and disclinations. Conversely, the *ζ* = 4.5 system shows a distinct relationship between melting pathway and defect evolution. Solid starts to melt after the appearance of disclinations, which leads the system to the CE regime. In CE window, disclination grows nearly linearly, similar to the *ζ* = 1.0 case, and obeys the lever rule.^36^ As a result, in *ζ* = 4.5 system, with the accumulations of dislocations and disclinations, the system loses its long-range bond-orientational order and quasi-long-range positional order, leading to a direct transition from HX to I. We note that the evolution pathway of *ζ* = 4.5 system is similar to hard pentagon case, which undergoes a one-step melting process.^2^

The vanishing of H phase is the signal of one-step melting scenario in *ζ* = 4.5 system. BKTHNY theory assumed that isolated dislocations dissociated from neutral dislocation pairs will destroy the quasi-long-ranged positional order and long-ranged bond-orientational order, driving the solid-to-H transition. This assumption is confirmed by previous investigation^37^ and *ζ* = 1.0 case in present work (Figure 3A). However, in *ζ* = 4.5 system (Figure 3C), although dislocations emerge from *ϕ*_*A*_ = 0.722, configurations still persist a quasi-long-range order of the HX phase until system starts to change to the I phase (*ϕ*_*A*_ = 0.719). This stable nature of configuration should be the essential reason for the emergent one-step melting behavior in *ζ* = 4.5 system.

Because the particle dynamics is closely correlated with the certain state of system, it is convenient to understand collective behavior of system through the kinetic information. Previous investigations^10^^,^^38^^,^^39^^,^^40^^,^^41^ revealed that the dynamics of particles exhibit a highly cooperative behavior even in hard particle systems. The main dynamical feature of high-symmetry particle is hopping-like motion, which is related to the cage structure formed by local environment of moving particles. Note that this local environment even includes the space of tens of particle length scales. Here, we define the time evolution of Lindemann parameter to investigate the dynamical behavior of particles, $L_{6}\left(t\right)=\frac{{\left\langle {\left|r\left(t+t_{0}\right)-{\left\langle r\left(t+t_{0}\right)\right\rangle }_{6}\right|}^{2}\right\rangle }^{1/2}}{a}$. Here, *t*_0_ is the starting time of a dynamical sequence, at which the neighbors of the reference particle are recognized, ***r***(*t*+*t*_0_) is the position of reference particle at time of *t*+*t*_0_, ⟨***r***(*t*+*t*_0_)⟩_6_ is the average position of the first 6 nearest neighbors of the reference particle (identified at time *t*_0_) and *a* is the ideal lattice constant of HX at certain density of configuration. The quantity *L*_6_(*t*) describes the evolution of cage structure around a reference moving particle, reflecting the particles’ escaping dynamics from its original cage structure. At *t* = 0, *L*_6_(0) is the Lindemann parameter that was discussed in the previous reports.^41^^,^^42^

We take *ϕ*_*A*_ = 0.730 in *ζ* = 1.0 system and *ϕ*_*A*_ = 0.720 in *ζ* = 4.5 system for comparison, where their configurations have a similar number of defects (see the inset colored configurations in Figures 3B and 3D; Table S3) but are in different states. At the beginning, *L*_6_(*t*) of *ϕ*_*A*_ = 0.720 configuration is larger than *ϕ*_*A*_ = 0.730 configuration due to its lower density nature (see inset in Figure 3E), which provides particles more space to vibrate. As time increases, *L*_6_(*t*) starts to grow, indicating the deformation of the cage structure and the preparation for inside particles to escape via hopping motion. This hopping behavior is a highly cooperative motion. The previous work^10^ showed that a particle migration requires fluctuation to lower the local density surrounding the migrating particle. Thus, the hopping behavior (or growth behavior of *L*_6_(*t*)) represents the stability of configuration against perturbations. In Figure 3E, though *L*_6_(0) of *ϕ*_*A*_ = 0.730 is smaller than *L*_6_(0) of *ϕ*_*A*_ = 0.720, its growth rate is significantly larger. Within very short timescale, the value of *L*_6_(*t*) of *ϕ*_*A*_ = 0.730 exceeds that of *ϕ*_*A*_ = 0.720 case and continues to grow with a much higher speed. This rapid increase of *L*_6_(*t*) in *ϕ*_*A*_ = 0.730 system demonstrates that its cage structure/configuration is fragile, which is consistent with its liquid nature. Conversely, the growth of *L*_6_(*t*) of *ϕ*_*A*_ = 0.720 is much slower, and it is hard to approach 1.0 (within our simulation time limitation for dynamics, 1.1×10^8^ MD steps, about 200 times of characteristic timescale *τ*_0_). This value represents the event that particles executed their first hopping activities. The previous trends in *L*_6_(*t*) evolution are consistent with the structural relaxation behaviors of two configurations, shown in Figure 3F, where the relaxation of *ϕ*_*A*_ = 0.720 is much slower than *ϕ*_*A*_ = 0.730, showing a more stable property. Qi et al.^43^ revealed that increasing stiffness of crystal will lead the crossover from a two-step transition to a first-order one-step transition in hard disk system, which supports our observations.

### Size-dependent melting process

**Figure 4:**
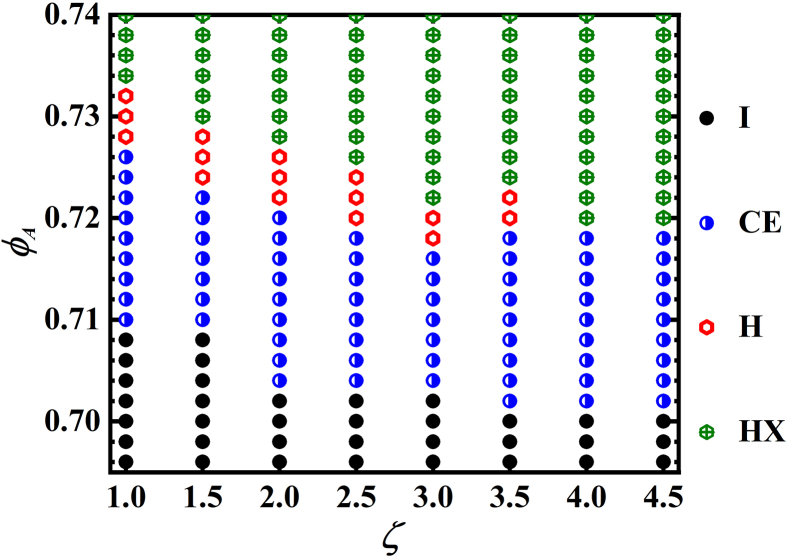
Summary of observed melting pathways of yolk-like particles Simulations are executed under temperature of 1.0 (ε/*k*_B_), the results of *ζ* ≤ 3.0 are obtained after 6×10^7^ MD steps, and results of *ζ* ≥ 3.5 are obtained after 1.5×10^8^ MD steps. HX, hexagonal crystal; H, hexatic phase; CE, coexistence; I, isotropic fluid.

Through a systematic investigation, we summarize the observed melting pathways of model particles in Figure 4. Particles with *ζ* ≤ 3.5 show a two-step melting scenario, including a continuous HX-to-H transition and a subsequent first-order H-to-I transition. Conversely, the particles of *ζ* ≥ 4.0 show a first-order HX-to-I transition. We notice that, in *ζ* = 3.5 system (see Figure S3), when the relaxation time of system is not long, e.g., 6×10^7^ and 1.0×10^8^ MD steps, its melting pathway is first-order HX-to-I transition. However, with a sufficient relaxation time, e.g., 1.5×10^8^ MD steps, melting behavior of system alters to the two-step scenario. This observation makes us consider that the observed first-order one-step transition is a non-equilibrium phase transition because though the model particle is yolk-like, the activity of internal core does not seem to influence the phase behavior of particles (see the tests on the particles without internal core of *ζ* = 1.0 and 4.5 in Figures S1 and S4 in SI), implying that the previous observation is a hard circular-particle based behavior, which should give a same melting behavior with hard disks in 2D due to it exhibits a similar excluded volume effect with hard disk. Moreover, the equilibrium melting behavior of hard disks is well-established as the two-step scenario.^23^^,^^24^^,^^25^ This emergent first-order one-step transition closely relates to the particularly stable nature of the structure, which represents a metastable state arrested by dynamics of large size particles in Langevin systems. We highlight that the duration time of observed one-step scenario in large size systems is due to our simulation time limitation (6.0×10^7^∼1.5×10^8^ MD steps in *ζ* = 4.5 system in our simulation, which is about 150 times of characteristic timescale of *τ*_0_).

## Discussion

**Figure 5:**
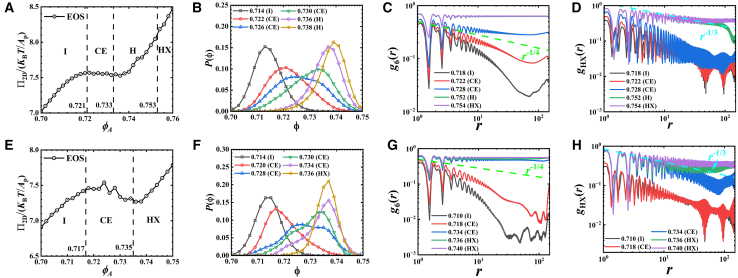
The melting pathways of simple hard disks with radii of *R*_0_ and 20*R*_0_, the friction constant of particle is proportional to its radius (A–D) The obseved melting pathway of simple hard disks with radius of *R*_0_ obtained from Langevin dynamics simulation after 1.0×10^8^ MD steps run. (E–H) The obseved melting pathway of simple hard disks with radius of 20*R*_0_ obtained from Langevin dynamics simulation after 1.0×10^8^ MD steps run.

Given that the interaction between model hard circular particles (the excluded volume effect) is similar to the interaction between hard disks, we investigate the universality of the dynamic-effect on hard-disk particles in 2D melting by performing additional independent Langevin dynamics simulations on simple hard disks. For each disk in simulation, its friction constant is set to be proportional to the radius. The results for particles with radii of *R*_0_ and 20*R*_0_ are shown in Figure 5, and they confirm that the dynamic-effect still works in these simple hard disk systems. The melting pathway of *R*_0_ particles is two-step scenario whereas the 20*R*_0_ particles is one-step, being consistent with our above coarse-grained model particle case. Here, the 20*R*_0_ case also shows a robust behavior, its one-step melting scenario observation is stable from 5.0×10^7^ to 2.0×10^8^ MD steps and even longer (beyond 310 times of characteristic timescale *τ*_0_, Figure S6).

The energy dissipation plays a crucial role in the emergence of one-step melting pathway of hard circular-particles/disks. For particles without energy dissipation, e.g., *NVT* ensembles using Andersen thermostat, tests show that the melting behavior of particles is independent of the particle size (see Figures S7 and S8). This result is consistent with the previous Monte Carlo^23^^,^^24^ and event-driven molecular dynamics^24^^,^^37^ simulations. Though the energy dissipation can induce the coexistence of HX and I phases of hard disks in non-equilibrium stationary state system,^36^ the mechanism of emerging HX and I coexistence in Langevin systems is different. In non-equilibrium stationary state system,^36^ the dissipation of particle between two external energy injections is the main source of the phase separation. However, in Langevin systems, under the limitation of fluctuation-dissipation theorem, the attached heat bath will continuously provide energy for each particle to compensate its dissipation. This suggests the occurrence of HX and I coexistence in Langevin systems is not solely due to the effect of energy dissipation but instead originates from the cooperative dynamical behavior of particles as discussed previously. Considering that increasing inertia (elastic collision) can effectively weaken the dissipation of the particles, it is also interesting to test the influence of inertia (elastic collision) on the dynamical behavior/phase transition of the system in the future.

In reality, the frictional force exerted on moving objects by the environment (e.g., air and water, etc.) are typically size-dependent. Thus, the effect discussed previously should be under consideration when analyzing their collective dynamics. This is particularly relevant for particles with size ranging from 10^−7^ to 10^−5^ meters, which undergo Brownian motion driven by thermal perturbations. This size range is significant, as it includes various elements essential to life activities (e.g., proteins, nucleic acids)^44^ and industrial production of low-dimensional micro- and nano-scale materials.^45^ Our findings are applicable to these fields.

Our further tests show that the emergence of one-step melting pathway depends critically on both particle size and system temperature. Smaller particle, such as *ζ* = 1.5 particle, still follows a two-step scenario even its friction constant is set to be as large as *ζ* = 4.5 particle (see Figure S2), while lower system temperature will induce the non-equilibrium one-step melting pathway (see Figure S1). These observations suggest an effective diffusion-dependent (i.e., *D*_eff_, or dynamic-effect) melting pathway of hard disks in Langevin systems.

### Conclusion

In conclusion, in Langevin system, we reveal that the observing melting transition of hard circular-particles/disks is dynamical relevant. In small size particle system, melting pathway obeys two-step scenario, including a continuous HX-to-H transition and a first-order H-to-I transition. In contrast, in large size particle system, we observe a non-equilibrium first-order HX-to-I transition. Further investigation reveals the emergent first-order melting pathway relates to the cooperative (cage) dynamics of particles, which can be induced in Langevin systems by increasing the size of particle and/or reducing the temperature of the system to slow down the effective diffusion behavior of particles. In larger size particle system, cage dynamics relax more slowly and configuration becomes stiffer. These findings reveal a novel melting phenomenon that a system might be trapped into a metastable state showing a non-equilibrium melting pathway, which distinguishes from the equilibrium melting behavior. The phase behavior of hard disks is a long-term debate until its determination ten years ago, due to the various melting transitions had been observed in investigations. Present work reveals that, during a melting process, system may show a non-equilibrium melting pathway that differs from equilibrium melting behavior, which provides a reasonable explanation for this long-running debate.

### Limitations of the study

In our model, we assumed that the friction constant of particle is proportional to the particle size (i.e., the radius of circular particle), such a law works in three-dimensional systems, there should be a different relationship between the friction constant and particle size in two-dimensional cases. Although the results presented in this work were obtained from Langevin dynamics simulations, their applicability in reality needs to be further verified through experimental works. In present work, we just systematically investigated the size-effect on the melting transition of 2D hard circular-particles/disks, their specific melting transition is also relevant to the temperature of system according to our tests (as mentioned in the main text), which is worth investigating in the future works. Moreover, the underlying mechanism of such effects is still undisclosed.

## Resource availability

### Lead contact

Requests for further information and resources should be directed to and will be fulfilled by the lead contact, Zhanglin Hou (zl_hou@tju.edu.cn).

### Materials availability

This study did not generate new unique reagents.

### Data and code availability

Data: All data reported in this paper will be shared by the lead contact upon request.

Code: This paper does not report original code.

Additional information: Any additional information required to reanalyze the data reported in this paper is available from the lead contact upon request.

## Acknowledgments

We thank Profs. Zhongyuan Lu, Zhaoyan Sun, and Youliang Zhu for the help on GALAMOST. Z.H. thanks Profs. Haim Diamant, Jinglei Hu, Mingcheng Yang, Dong Zhao, and Huaping Li for useful discussions. Z.H. and B.Z. acknowledge Prof. Fangfu Ye for the support on computing resources. This project supported by the 10.13039/501100001809National Natural Science Foundation of China (grant 12104453 to Z.H.; grant 22273067 to L.H.; and grants 12274098 and 12250710127 to S.K.).

## Author contributions

Z.H., S.K., and L.H. conceived the project. Y.Z., S.J., and J.W. performed the simulations. Y.Z., X.S., and X.H. helped on algorithms and codes. Y.Z., S.K., and Z.H. analyzed the data. Y.Z., S.K., L.H., B.Z., and Z.H. interpreted the data with help from all other authors. Z.H., Y.Z., L.H., B.Z., X.S., X.H., and S.K. wrote the manuscript.

## Declaration of interests

The authors declare no competing interests.

## Declaration of generative AI and AI-assisted technologies in the writing process

During the preparation of this work, the author(s) used DeepSeek to improve the readability of the English text. After using this tool or service, the author(s) reviewed and edited the content as needed and take(s) full responsibility for the content of the publication.

## STAR★Methods

### Key resources table

**Table:** 

REAGENT or RESOURCE	SOURCE	IDENTIFIER
**Software and algorithms**		

Galamost4.0.2	Y.-L. Zhu et al.^26^^,^^27^	https://docs.hpc2n.umu.se/software/apps/GALAMOST/
Intel C++ Compiler	Intel Corporation	https://www.intel.com/content/www/us/en/developer/tools/oneapi/dpc-compiler.html#gs.mz41zg

### Method details

#### Simulation details

For yolk-like particles, we build particles using coarse-grained model by putting truncated 12-6 Lennard-Jones beads on the inside core and outside hollow ring. The core is constructed by putting the centers of two beads with radius of *R*_c_=2∗*R*_0_ on the center of mass of core. The two beads are overlapped to form a disk-like core, as the requirements on building of rigid body in GALAMOST. The hollow ring is built by putting beads on the arc of ring with a distance around 0.5σ between two adjacent beads, i.e., for a hollow ring with radius of *R*_r_, the beads are put on the arc of ring with radius of *R*_r_-*R*_0_, with distance of $\frac{2\pi \left(R_{r}-R_{0}\right)}{\left\lceil 2\pi \left(R_{r}-R_{0}\right)/0.5\sigma \right\rceil }$, where the *R*_0_=2^-5/6^σ is the radius of bead on the ring, and the ⎡…⎤ means taking the integer upwards from the float result. The interaction between beads are controlled by Weeks-Chandler-Andersen (WCA) potential, which is defined as

(Equation 2) $$u\left(r_{ij}\right)=\left\{ \begin{array}{c} 4\varepsilon \left[{\left(\frac{A\sigma }{r_{ij}}\right)}^{12}-{\left(\frac{A\sigma }{r_{ij}}\right)}^{6}\right]+\varepsilon ,r_{ij}\leq 2^{1/6}A\sigma \\ 0,\;r_{ij}>2^{1/6}A\sigma \end{array}\right.\text{,}$$

where *r*_ij_ is the distance between beads, ε and σ are the characteristic (unit of) energy and distance, respectively. *A* is control parameter due to the different radii of beads on the core and ring, for the interaction between beads on the rings, *A*=1.0, and for the interaction between a bead on the ring and a bead on the core, *A*=1.5. In order to shorten the buffer distance between beads, we set ε=100*k*_B_*T*, which can better model the hard interaction between particles. The masses of ring and core are both set to be 1.0, and the friction constant of each bead is set to be 1.0.

For the simulation of simple hard disks, we take a single disk as each particle with interaction of Equation 2 between disks. We adjust the radius of particle by tuning the value of *A*, e.g., for particle with radius of 20*R*_0_, *A* is 20. The mass of each disk is 1.0. Here, the friction constant of each disk is set to be *A*.

Simulations are performed using a GPU-based Molecular Dynamics (MD) package, the GPU-Accelerated Large-scale Molecular Simulation Toolkit (GALAMOST) and homemade codes. We use GALAMOST to simulate the Brownian dynamics of yolk-like particles, and for the simulations of Brownian dynamics and *NVT* ensembles (using Andersen thermostat) of simple hard disks, we use our homemade CPU-based MD codes. Initial configurations are crystalline states, there *N*=128×128 (for simple disk systems), 250×250 (for yolk-like systems) or 400×400 (for finite-size-effect test) particles are put in the simulation box on a perfect hexagonal lattice structure, whose lattice constants are tunable to match with the box under certain density of system, *ϕ*_*A*_=*NA*_p_/*L*_*x*_/*L*_*y*_, where *A*_p_ is the area of particle, *L*_*x*_ and *L*_*y*_ are the lengths of box on *x*-axis and *y*-axis, respectively. The relationship between *L*_*x*_ and *L*_*y*_ is fixed to be $L_{x}/L_{y}=2/\sqrt{3}$ due to the hexagonal crystal. In simulations, in yolk-like particle systems, there at least 6×10^7^ MD steps (with unit of 0.005×($\sqrt{m\sigma^{2}/\varepsilon }$)) are run and the last 10^7^ MD steps with configurations recorded every 10^5^ MD steps for statistical analysis, and in simple disk systems, there totally 10^8^ MD steps are run and the last 10^7^ MD steps with configurations outputted every 10^5^ MD steps for statistical analysis, this ensures that each obtained result is calculated by averaging 100 configurations. To analyze the dynamics of selected systems, we take the equilibrium structure as initial configuration and run additional 2.1×10^7^ MD steps with configurations recorded every 10^4^ MD steps, the last 2.0×10^7^ MD steps will be used for analysis of dynamics. The results presented in current work are based on the observations of *N*=250×250 (for yolk-like systems) and 128×128 (for simple disk systems) particles systems. We mention that our simulation system is large enough and the finite-size effect is negligible by comparing with previous investigations^2^^,^^5^^,^^7^ and our tests (Figures S4 and S5).

In simulations, there will be some size-changing effects when we adjust the size of the coarse-grained circular particle and the simple hard disk. Increasing the size of the coarse-grained circular particle, the outline of particle becomes smoother and the interparticle interaction becomes ‘harder’, which leads the particle to be more approximate to a hard disk, while a simple disk will become ‘softer’ when its size increases. However, these effects will not influence our conclusion of the present work because the softness of interaction between particles will not change the two-step melting scenario nature of hard circular-particles/disks.^46^

### Quantification and statistical analysis

#### Order parameters and correlation functions

We determine the melting behavior of systems by a combination of order parameters including 6-fold bond-orientational order and positional order with hexagonal lattice, susceptibility of bond-orientational order, local density distributions, as well as spatial correlation functions of bond-orientational order and positional order.

The 6-fold bond-orientational order is defined as

(Equation 3) $$\Psi_{6}e^{i\omega }=\left\langle \phi_{6}\left(r_{j}\right)\right\rangle \text{,}$$

where *ω* denotes global phase, ⟨…⟩ denotes ensemble average, and $\phi_{6}\left(r_{j}\right)=\frac{1}{N_{j}}\sum_{k=1}^{N_{j}}e^{i6\theta_{jk}}$, here, *N*_*j*_ is the number of neighbors of particle *j*, and *θ*_*jk*_ is the angle between an arbitrary fixed axis and the line connecting the centers of particles *j* and *k*, the *N*_*j*_ and corresponding neighbors are obtained through Voronoi construction.

The positional order relates to hexagonal lattice *S*_HX_ is defined as

(Equation 4) $$S_{\text{HX}}=\left|\frac{1}{N}\sum_{j=1}^{N}\xi \left(r_{j}\right)\right|\text{,}$$

where $\xi \left(r_{j}\right)=e^{iG\cdot r_{j}}$ with ***G*** of the reciprocal lattice of HX crystal.

The susceptibility of 6-fold bond-orientational order is defined as

(Equation 5) $$\chi_{6}=N\left(\left\langle \Psi_{6}^{2}\right\rangle -{\left\langle \Psi_{6}\right\rangle }^{2}\right)\text{,}$$

and the spatial correlation function of bond-orientational order/positional order is defined as

(Equation 6) $$g_{6/\text{HX}}\left(r\right)=\text{Re}\left\langle v^{*}\left(r_{j}\right)v\left(r_{j}+r\right)\right\rangle \text{,}$$

where Re represents the operator returning the real part of the result value, and *v* denotes the $\phi_{6}$ or ξ.

The self-part intermediate scattering function (self-ISF) is defined as

(Equation 7) $$F_{s}\left(k,t\right)=\frac{1}{N}\left\langle \sum_{j=1}^{N}e^{-ik\cdot \left(r_{j}\left(t\right)-r_{j}\left(0\right)\right)}\right\rangle \text{,}$$

where wave vector *k* is set to be 2π/*R*_ch_ with *R*_ch_ the length scale of the first (maximum) peak of the radial distribution function.

The equation of state of system is determined by calculating the pressure in the system, which consists of an ideal-gas part $P^{\text{id}}$ and an “internal virial” part $P^{\text{in}}$^47^:

(Equation 8) $$P=P^{\text{id}}+P^{\text{in}}=\rho k_{B}T+W/S\text{,}$$

where *ρ* is the number density of system, *S* is the area of system (in two dimension), and $W=\frac{1}{D}\sum_{i=1}^{N}r_{i}\cdot f_{i}$ with *D* the dimension of system.

For all configurations, the local density distribution is calculated by averaging over a circular region with a radius of 30*R*_p_, where *R*_p_ represents the radius of particle, e.g., for yolk-like particle, *R*_p_=*R*_r_, and for simple disk particle, *R*_p_=*AR*_0_. The Voronoi area of individual particle is first calculated, and subsequently the local density is obtained through the ratio between particle area and Voronoi area within local region.

We determine the coexistence (CE) windows based on the local density distribution of configurations. For each CE state, we carefully checked that there are two distinguishable phases contained in a single configuration, corresponding to a high-density phase and a low-density phase. Theoretically, it should be more precise if we determine the CE window using Maxwell construction, however, its result is sensitive to the size of system that a smaller size system will provide a much wider CE region.^23^^,^^43^ As a consequence, considering the size limitation of our systems, we adopt the method described above to determine the boundaries of CE state, the more precise boundaries can be obtained in the future using larger size systems through Maxwell construction.

The topological defects including neutral, dislocation and disclination are determined by using Voronoi tessellation of the centers of mass of particles, following the operations in previous investigations.^1^^,^^2^ For each particle, we count the number of neighbors that is obtained through Voronoi construction to determine its disclination charge by the definition of $q_{j}=n_{j}-6$, where $n_{j}$ is the number of neighbors of particle *j*. Charged particles with $q_{j}\neq 0$ were picked out for further analysis. Considering that they are often not clearly separated but agglomerate into clusters, any two charged particles will be treated within a cluster if they are Voronoi neighbors with each other. For each defect cluster, if its total disclination charge $q=\sum_{j}q_{j}$ is not 0, we define it as disclination defect, otherwise, if the cluster with q=0, we further calculate its total Burgers vector $b=\hat{z}\times \sum_{j}q_{j}r_{j}$ according to the disclination charges and positions of particles within the cluster. Here, $\hat{z}$ is the unit vector along the out-of-plane axis and $r_{j}$ is the position vector of particle *j*. By snapping the total Burgers vector to its closest lattice point in a hexagonal lattice, which is the ideal crystalline lattice at certain density of configuration, we define the cluster with q=0 and b≠0 as dislocation defect and the one with q=0 and b=0 as neutral defect.
