# Supplementary material for: Observing one-step melting pathway in two-dimensional hard circular particle system
Source: iScience. 2025 Jul 12;28(8):113107. doi: 10.1016/j.isci.2025.113107 (PMC12312111; doi:10.1016/j.isci.2025.113107)
Supplement: Document S1. Figures S1–S8 and Tables S1–S3 [file mmc1.pdf]

iScience, Volume *28*

Supplemental information

Observing one-step melting pathway in two-dimensional hard circular particle system

Yanwen Zhu, Shenhua Jiang, Jieli Wang, Xin Song, Xing Huang, Bin Zheng, Linli He, Shigeyuki Komura, and Zhanglin Hou

Table S1 The number of beads to build the ring of yolk-like particles in simulations.

ζ	Number of beads (N_{bead})
1.0	22
1.5	29
2.0	36
2.5	43
3.0	50
3.5	57
4.0	64
4.5	71

Table S2 The characteristic time τ_0 of each ζ particle. τ_0 is the time scale that a particle diffuses one particle length scale, i.e., $\langle \Delta r^2(\tau_0) \rangle = 4R_r^2$.

ζ	τ_0 ($\times 10^3$ MD steps)
1.0	25
1.5	50
2.0	88
2.5	142
3.0	210
3.5	298
4.0	409
4.5	550

Table S3 Fractions of defects in ϕ_A=0.730 (H phase) of ζ=1.0 system and ϕ_A =0.720 (HX phase) of ζ=4.5 system.

ζ	ϕ_A (Phase)	Neutral	Dislocation	disclination
1.0	0.730 (H)	0.023%	0.017%	0.00314%
4.5	0.720 (HX)	0.021%	0.0133%	0.00184%

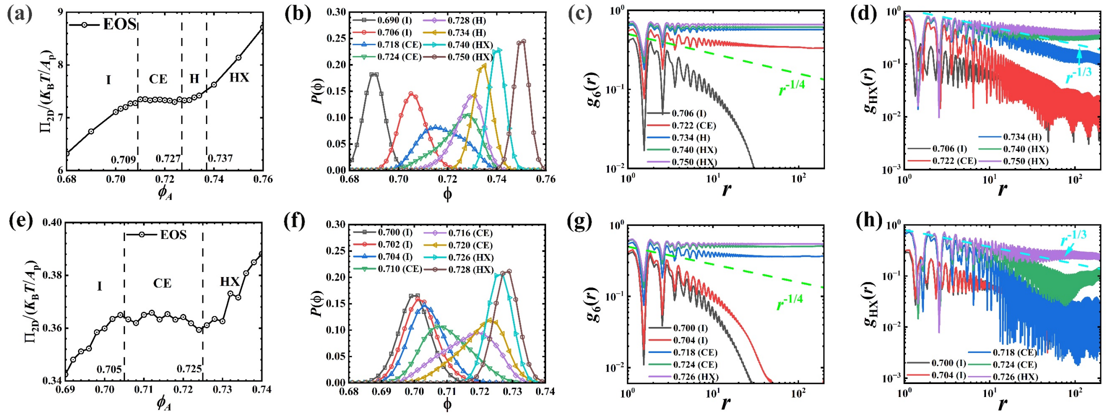

Figure S1 Melting pathways of ζ=1.0 coarse-grained yolk-like particles without internal core under temperature of 1.0 (a-d) and 0.05 (e-h), respectively. Results obtained after 6×10^7 (a-d) and 1.5×10^8 (e-h) MD steps run using N=250×250 particles.

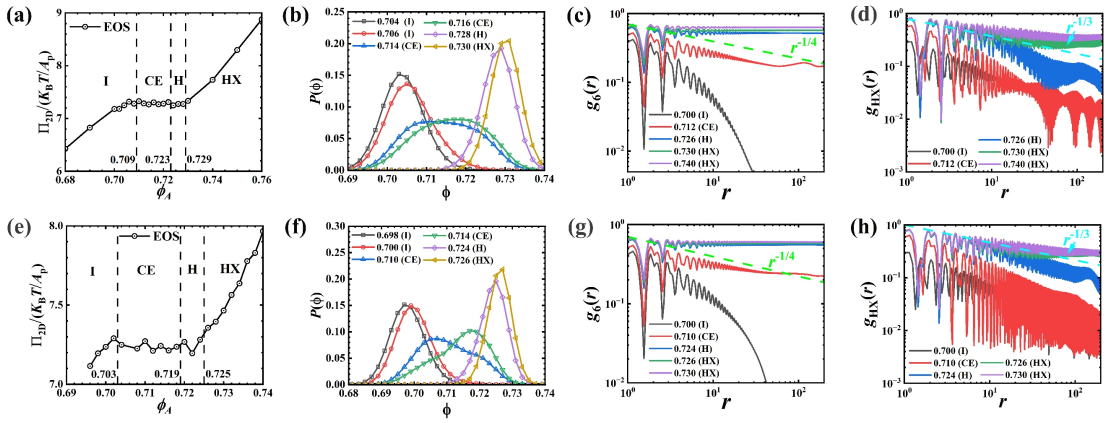

Figure S2 Melting pathways of ζ=1.5 coarse-grained yolk-like particles with N_{bead}=29 (a-d) and N_{bead}=71 (e-h), respectively. Results obtained after 6×10^7 (a-d) and 1.5×10^8 (e-h) MD steps run with temperature of 1.0 using N=250×250 particles. Note that the N_{bead}=71 of ζ=1.5 particle shows a similar single particle behavior with ζ=4.5 particle, i.e., the same diffusion constant, because they have the same number of beads (friction constant). The ζ=1.5 particles constructed using N_{bead}=71 are only used to test the size-effect of particle on the melting pathway. The results reported in the main text and the other parts in this Supplementary Information are based on the particles described in Table S1.

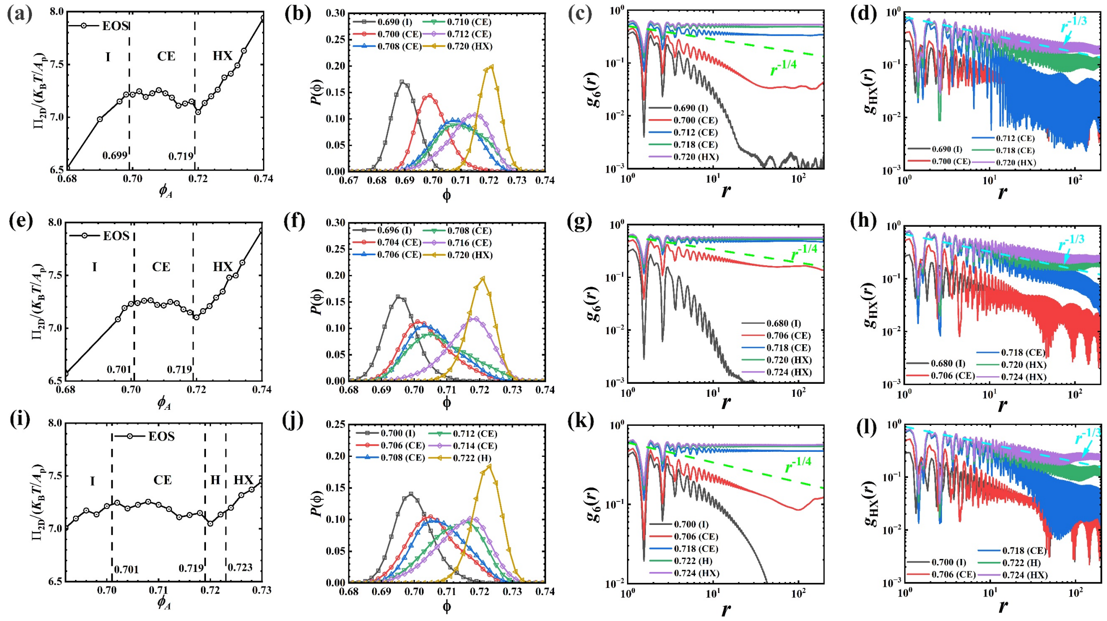

Figure S3 Melting pathways of ζ=3.5 coarse-grained yolk-like particles obtained after 6×10^7 (a-d), 1.0×10^8 (e-h) and 1.5×10^8 (i-l) MD steps run, respectively. Simulations are executed under temperature of 1.0 using N=250×250 particles.

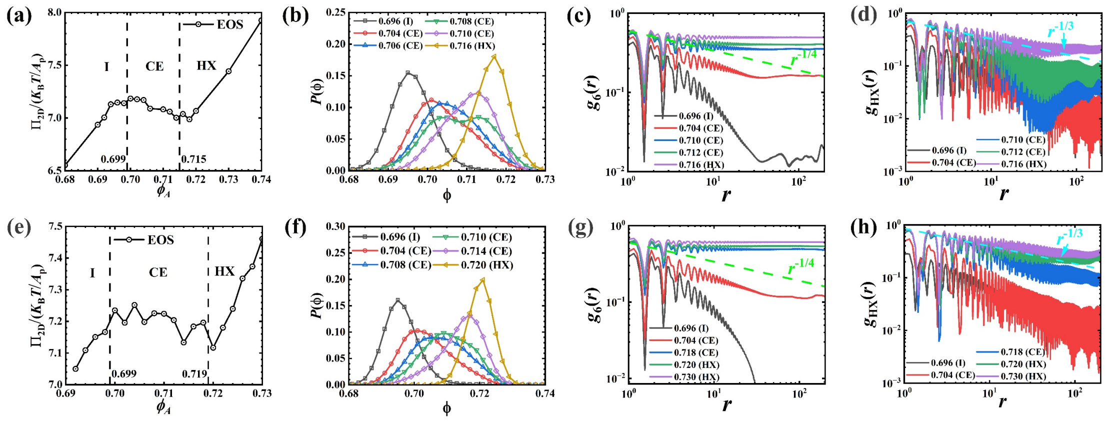

Figure S4 Melting pathways of ζ=4.5 coarse-grained yolk-like particles with (a-d) and without (e-h) internal core obtained after 6.0×10^7 (a-d) and 1.5×10^8 (e-h) MD steps run, respectively. Simulations are executed under temperature of 1.0 using N=250×250 particles.

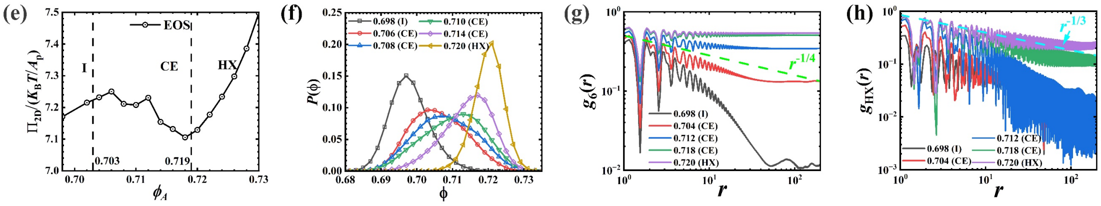

Figure S5 Melting pathways of ζ=4.5 coarse-grained yolk-like particles using N=400×400 particles. Results obtained after 1.5×10^8 MD steps run with temperature of 1.0.

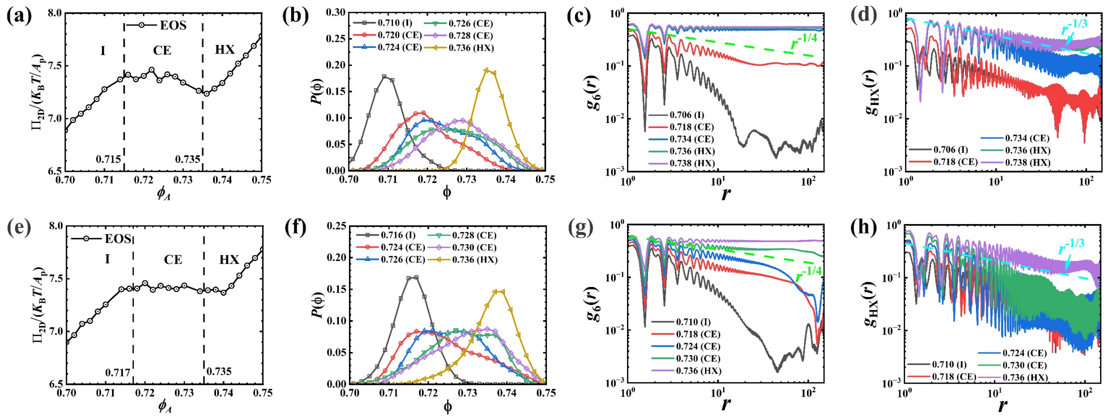

Figure S6 Melting pathways of simple hard disks with radii of 20R_0 obtained after 5×10^7 and 2×10^8 MD steps run, respectively. Simulations are executed under temperature of 1.0 using N=128×128 particles.

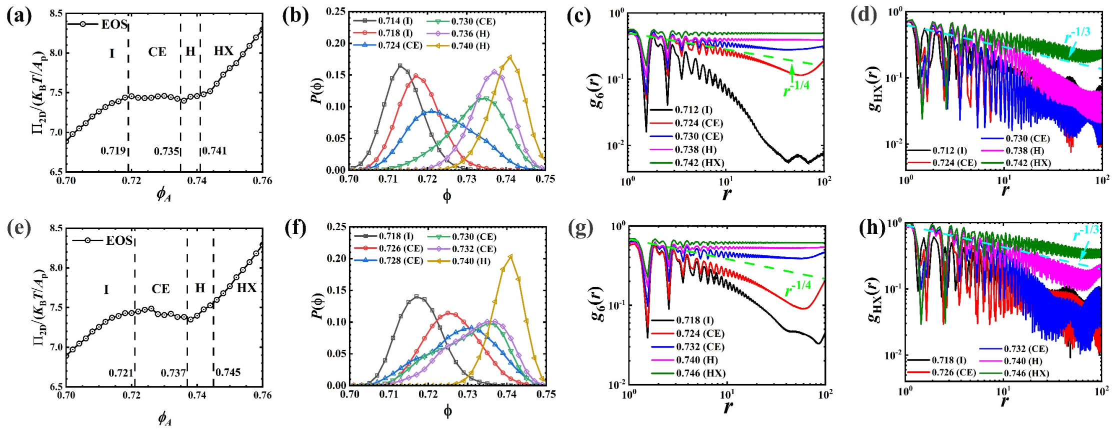

Figure S7 Melting pathways of simple hard disks with radii of 20*R*0 (a-d) and 50*R*0 (e-h) obtained from *NVT* ensembles using Andersen thermostat. Results obtained by 1.0×10^8 MD steps run with temperature of 1.0 using N=128×128 particles.

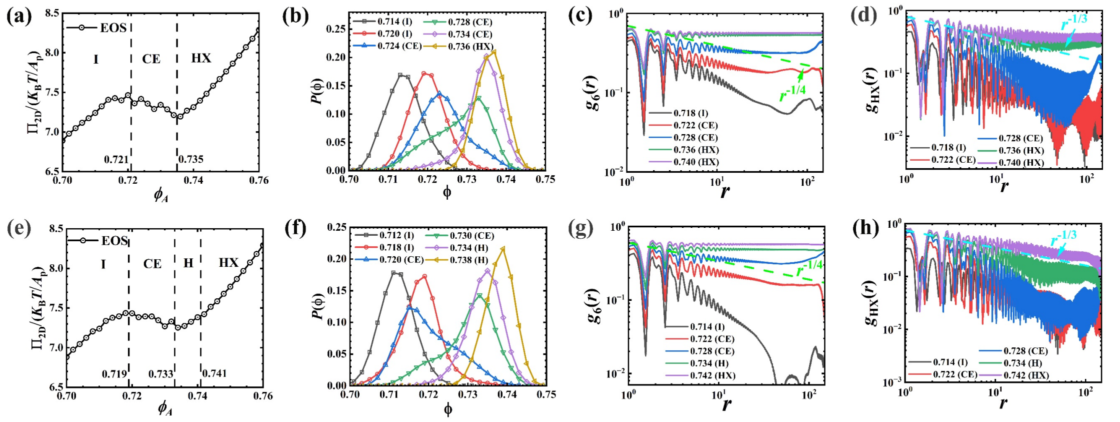

Figure S8 Melting pathways of simple hard disks with radius of 100R_0 obtained from Langevin dynamics simulation (a-d) and *NVT* ensemble using Andersen thermostat (e-h). The friction constant of particle in Langevin dynamics is 1.0. These two systems show a similar single particle behavior, i.e., overlapped MSD curves. Results obtained by 1.0×10^8 MD steps run with temperature of 1.0 using N=128×128 particles.
